# The admissibility of digital evidence from open-source forensic tools: Development of a framework for legal acceptance

**DOI:** 10.1371/journal.pone.0331683

**Published:** 2025-09-12

**Authors:** Isa Ismail, Khairul Akram Zainol Ariffin

**Affiliations:** 1 Pharmacy Enforcement Division, Petaling Jaya, Malaysia; 2 Center for Cyber Security, Universiti Kebangsaan Malaysia, Bangi, Malaysia; Florida International University, UNITED STATES OF AMERICA

## Abstract

The proliferation of cybercriminal activities from 2023 to 2025 has highlighted the critical role of digital forensics in legal proceedings; however, resource constraints often limit access to effective investigative capabilities. Despite the technical adequacy of open-source digital forensic tools, courts typically favor commercially validated solutions because of the absence of standardized validation frameworks for open-source alternatives, creating unnecessary financial barriers to high-quality forensic investigations. This study aims to validate and enhance the conceptual open-source digital forensic framework developed by Ismail et al. (2024) to ensure the legal admissibility of evidence acquired through open-source tools. Through a rigorous experimental methodology utilizing controlled testing environments, we conducted comparative analyses between commercial tools (FTK and Forensic MagiCube) and open-source alternatives (Autopsy and ProDiscover Basic) across three distinct test scenarios: preservation and collection of original data, recovery of deleted files through data carving, and targeted artifact searching. Each experiment was performed in triplicate to establish repeatability metrics, with error rates calculated by comparing the acquired artifacts with control references. Our findings demonstrate that properly validated open-source tools consistently produce reliable and repeatable results with verifiable integrity comparable to their commercial counterparts. The enhanced three-phase framework integrating basic forensic processes, result validation, and digital forensic readiness to satisfy Daubert Standard requirements while providing practitioners with a methodologically sound approach. This study contributes significantly to digital forensics by democratizing access to forensically sound investigative capabilities without compromising legal admissibility requirements, ultimately benefiting resource-constrained organizations while maintaining the evidentiary standards necessary for judicial acceptance.

## Introduction

The digital space has recorded a notable increase in cybercriminal activities from 2023 to 2025, making it difficult for law enforcement agencies worldwide. The Australian Government report showed that 47% of the respondents encountered at least one type of cybercrime within a year, and the most common types included online abuse (27%), malware attacks (22%), identity crimes (20%) and fraud (8%) [1]. The study also indicated that 34% of the respondents had been victims of data breaches, yet most of these cases were not reported to the authorities, exposing a major deficiency in the official statistics of cybercrime. The expansion of the Internet of Things (IoT) ecosystem will drive cybercrime rates to grow by 15-20% annually through 2025 because it provides new attack vectors to cybercriminals [2].

Digital forensics has become an essential discipline that deals with methodologies, techniques, and tools used in the identification, collection, preservation, and analysis of digital evidence for legal purposes [3]. The field has expanded to address eight primary focus areas: basic theory and methods, physical equipment and forensic methods, image forgery identification, file recovery and data extraction, smartphone and social network forensics, case-based forensics, automatic identification technology, and cloud forensics [4]. This multifaceted approach enables investigators to deal with different kinds of digital crimes, as well as to preserve evidence quality throughout the investigation lifecycle. The “Forensic of Things” paradigm that is emerging extends these capabilities to IoT environments because traditional forensic approaches are not sufficient in IoT due to device diversity, proprietary communication protocols, and data volume challenges [5].

Digital forensic procedures follow a standardized process to guarantee that the evidence is admissible. This process starts with the identification of potential digital evidence sources, followed by the preservation of the digital crime scene, collection by forensically sound methods, examination while maintaining the chain of custody, analysis to determine relevance, and presentation in court-admissible formats [6]. Each stage must adhere to strict protocols to avoid evidence modification or loss, which may render the findings inadmissible in legal proceedings. The methodological rigor of these procedures, as observed in the study by Edward et al. [7], affects judicial acceptance of digital evidence, thereby emphasizing the essential connection between technical processes and legal requirements.

Digital evidence is a vital element in cybercrime prosecutions, because it includes digital information that has probative value in legal proceedings. Such evidence is admissible if it can be proven authentic, reliable, complete, and in a good chain of custody [8]. Current developments in different jurisdictions show a growing trend to standardize the retrieval and protection of digitally stored information to enhance legal validity. This trend is a manifestation of the fact that digital evidence has its own set of problems that are different from physical evidence, thus necessitating specialized measures to ensure that it is not compromised during the investigative process [9].

The use of international standards is important for the development of uniformity in handling digital evidence. The ISO/IEC 27037:2012 standard provides detailed guidance for the identification, collection, acquisition, and preservation of digital evidence, whereas the ISO 27050 series address electronic discovery processes [10,11]. These standards provide a comprehensive description of the entire lifecycle of digital evidence management, from identification to preservation, analysis, and presentation. A comparison of these standards shows that there are complementary approaches that result in the development of effective frameworks for the admissibility of digital evidence across jurisdictions when properly integrated. The reason for standardization is even more significant in light of the fact that cybercrime has no borders, and investigations and prosecutions often require cross-jurisdictional collaboration.

Two main categories of tools are used in digital forensic investigations: commercial solutions and open-source alternatives. Commercial tools developed by specialized forensic software companies typically offer comprehensive feature sets, regular updates, dedicated support, and certification for legal proceedings. Two of the most widely used commercial platforms are EnCase by Guidance Software and FTK by AccessData, both of which have been widely used in courts. However, these tools often have substantial licensing costs, which may limit the accessibility of smaller law enforcement agencies or independent investigators [12]. In contrast, open-source digital forensic tools offer cost-effective alternatives with transparency in their underlying codes, allowing for peer review and validation of methodologies [13]. Autopsy, the Sleuth Kit, and CAINE offer robust capabilities but do not come with the financial constraints of commercial alternatives. The main challenge faced by open-source digital forensic tools in court proceedings is the issue of admissibility, mainly because of concerns regarding reliability, maintenance, and the lack of formal certification processes [14]. Multiple recent studies evaluating commercial versus open-source forensic tools have produced inconsistent results regarding tool efficiency, precision, and complete functionality. Studies that analyze these tool categories during forensic investigations show that commercial tools offer better user interfaces and workflow integration; however, open-source solutions demonstrate comparable or superior performance in particular forensic applications [15]. The Indonesian Supreme Court’s case file handling system through information technology adoption shows digital solutions gaining judicial acceptance, which creates potential opportunities for open-source forensic tools to gain wider legal use [16]. The digital supply chain models implemented in Industry 4.0 demonstrate how standardized digital processes improve transparency and trust, which could be applied to open-source forensic frameworks [17].

The technical sufficiency of open-source forensic tools does not eliminate the critical need for forensic frameworks to validate the evidence from open-source digital forensic tools for legal admissibility. Legal systems tend to choose commercially validated solutions because they lack standardized validation procedures for open-source alternatives even though these solutions do not have inherent technical limitations. This preference establishes unnecessary financial obstacles for top-quality forensic investigations and restricts new developments within the field [18].

Additionally, Carrier (2009) [30] discussed the admissibility of digital evidence requirements based on The Daubert Standard. The Daubert Standard is a legal standard used in the United States to determine the admissibility of scientific evidence in a court of law. In the context of digital forensics, the Daubert standard is used to assess the reliability and validity of digital forensic evidence such as computer logs, images of storage media, or other types of digital data.

The Daubert standard is based on the 1993 US Supreme Court case. It was used to determine whether the presented scientific evidence was relevant and reliable. When applying the Daubert standard in the context of digital forensics, the court considered the following factors:

- Testability: The methods used to produce evidence must be testable and capable of independent verification.
- Peer Review: The methods used must have been subject to peer review and publication, indicating that they have been subjected to scrutiny by the scientific community.
- Error Rates: The methods used must have established error rates or be capable of providing accurate results.
- General Acceptance: The methods used must be widely accepted by the relevant scientific community.

By considering these factors, the court can determine whether the digital evidence presented is reliable and credible to support the prosecution or defense’s case. Therefore, this study aimed to validate the conceptual open-source digital forensic framework proposed by Ismail et al. (2024) [3] and improve it to ensure that the open-source tools can be applied and the evidence retrieved through it is admissible in court. This study employed a rigorous experimental methodology to validate and enhance the conceptual framework developed by Ismail et al. (2024) [3] to ensure the legal admissibility of digital evidence acquired through open-source forensic tools. The validation approach utilized a controlled testing environment with two windows-based workstations, implementing a comparative analysis between commercial tools and open-source alternatives. The experimental design incorporated three distinct test scenarios: preservation and collection of original data, recovery of deleted files through data carving, and targeted artifact searching in case-specific scenarios. Following methodologies from Flavien (2014) [28] and NIST Computer Forensics Tool Testing standards, each experiment was conducted in triplicate to establish repeatability metrics, with error rates calculated by comparing the acquired artifacts to control references.

This study makes significant contributions to digital forensics by validating and improving a framework that ensures the legal admissibility of evidence obtained through open-source digital forensic tools. Through rigorous experimental validation that compares open-source tools with commercial alternatives in three distinct test scenarios, this study addresses a critical gap in the forensic science literature, where cost-effective alternatives have remained underutilized despite comparable technical capabilities. The developed three-phase framework, which integrates basic forensic processes, result validation, and digital forensic readiness, provides practitioners with a methodologically sound approach that satisfies the requirements of the Daubert standard for forensic evidence. By empirically demonstrating that tools such as Autopsy and ProDiscover consistently produce reliable and repeatable results with verifiable integrity, this study challenges the preference for costly commercial solutions in legal proceedings. For resource-constrained organizations, the framework offers practical implementation guidance while maintaining evidentiary standards. Integration of digital forensic readiness planning further enhances organizational capability to effectively deploy open-source solutions. This study ultimately democratizes access to forensically sound investigative capabilities without compromising legal admissibility requirements, thus benefiting both academic and practitioner communities globally.

The remainder of this paper is organized as follows. The next section discusses the capability of open-source digital forensic tools compared to commercial tools. This is followed by the methodology section, in which evidence sample preparation, experimental instrument selection, test framework, set the rule, and outline the error rate. The results of the three experiments on the evidence samples are presented, followed by a discussion. The discussion covers the factors that affect the admissibility of digital evidence produced by open-source digital forensic tools. The improved open-source digital forensic tool framework is presented next, followed by its readiness and discussion of its advantages and disadvantages. Finally, the conclusions are presented in the final section.

## The capability of open-source digital forensic tools compared to commercial tools

Digital forensic tools play a pivotal role in cybersecurity investigations, enabling practitioners to collect, analyze, and present digital evidence. As cyber threats grow in sophistication and diversity, the demand for effective forensic tools in law enforcement, corporate security, and academic settings has intensified. This section provides a comprehensive comparison between open-source and commercial digital forensic tools and examines their capabilities, limitations, and suitability for various investigative scenarios.

The field of digital forensics has evolved significantly over the past decade, driven by technological advancements and increasingly sophisticated cyber-threats. This evolution has given rise to a diverse ecosystem of forensic tools, broadly categorized as either open-source or commercial solutions. Open-source tools are freely available with accessible source code that can be modified and distributed by the community, whereas commercial tools are proprietary solutions developed and sold by specialized vendors. This distinction has substantial implications for forensic practitioners in terms of cost, capability, support, and legal admissibility of evidence.

The current digital forensic landscape encompasses tools designed for various specialized domains, including disk forensics, memory analysis, network forensics, mobile device investigation, and increasingly, Internet of Things (IoT) forensics. Recent comparative studies indicate that both open-source and commercial tools have evolved to address complex forensic challenges using different approaches and strengths [19]. The selection between these tool categories often depends on specific investigation requirements, organizational constraints, and examiner preferences rather than the inherent superiority of either approach.

Open-source digital forensic tools have attracted significant attention in recent years because they offer cost-effective alternatives to commercial solutions. The Sleuth Kit (TSK) and its graphical interface, Autopsy, represent one of the most comprehensive open-source forensic platforms, providing robust capabilities for disk image analysis and artifact examination. Recent studies demonstrate that Autopsy performs effectively in identifying and analyzing forensic artifacts within test images, successfully retrieving most available digital evidence across various scenarios [20]. The modular architecture of the platform facilitates plugin development and enhances its functionality for specialized forensic tasks.

For memory forensics, Volatility stands as a prominent open-source solution, enabling investigators to analyze RAM dumps and extract valuable evidence from volatile memory. This capability has become increasingly important because sophisticated threats often operate exclusively in memory to avoid detection. The ability of volatility to examine process information, network connections, and malware artifacts makes it an essential tool for comprehensive digital investigations. Recent developments have further enhanced memory forensic capabilities through specialized integrations, such as the MemoryIntegrator plugin for Autopsy, which seamlessly harmonizes with the Volatility framework to enable forensic analysts to identify and extract volatile memory from small-scale digital devices [21].

In the network forensics domain, open-source tools such as Suricata demonstrate impressive capabilities in intrusion detection and traffic analysis. Suricata’s multiprocessing configuration and load balancing make it particularly effective for analyzing traffic in both wired and wireless networks. This tool has been successfully deployed in IoT network forensic setups, effectively identifying threats and providing alerts for various attack scenarios, including Denial of Service and Man-in-the-Middle attacks [22].

Despite their substantial capabilities, open-source forensic tools have certain limitations. Comparative study has revealed that tools like Autopsy may struggle with advanced anti-forensic techniques, particularly when confronted with encrypted steganography [21]. This limitation highlights the need for complementary tools or techniques to handle complex investigations. Additionally, open-source tools may lack the polished user interfaces and comprehensive documentation that characterize their commercial counterparts, potentially increasing the learning curve for new users and requiring greater technical expertise for effective deployment.

Commercial digital forensic tools offer integrated solutions with professional support and regular updates that address the full spectrum of digital investigation requirements. EnCase and Forensic Toolkit (FTK) represent two of the most established commercial platforms that provide comprehensive capabilities for evidence acquisition, analysis, and reporting. These tools support various file systems, operating systems, and digital artifacts, with optimized workflows designed to enhance investigator efficiency. Comparative studies indicate that commercial tools often excel in advanced analytical capabilities, particularly in handling complex file systems and recovering deleted or fragmented data [19].

In mobile forensics, commercial solutions such as Cellebrite’s UFED Physical Analyzer, Belkasoft X, and Magnet Axiom offer sophisticated capabilities for extracting and analyzing data from smartphones and tablets. These tools support multiple extraction methods, including logic, file systems, and physical acquisition, and provide investigators with flexible options based on device accessibility and investigation requirements. Commercial mobile forensic tools typically maintain extensive device databases, ensuring compatibility with a wide range of mobile devices and operating systems [23,24].

Forensic Explorer, another notable commercial offering, has demonstrated robust performance in analyzing forensic images and handling various types of digital evidence. Recent comparative studies between Forensic Explorer and Autopsy TSK revealed that both tools exhibited strong performance in identifying and analyzing forensic artifacts, though with different strengths in specific scenarios. This finding underscores the importance of tool selection based on specific investigation requirements rather than general categorization as open source or commercial [20].

Although commercial tools offer comprehensive capabilities, they have certain limitations. Their proprietary nature can create dependency on vendor support and limit customization options. Moreover, the significant costs associated with commercial tools may be prohibitive for smaller organizations or practitioners. Updates and licensing fees represent ongoing expenses that must be factored into total ownership cost. In addition, proprietary algorithms and closed-source codes can raise questions about transparency and verifiability, potentially affecting the admissibility of evidence in legal proceedings.

Comparative analyses of open-source and commercial forensic tools have revealed significant performance differences across various forensic tasks. In a study evaluating EnCase, FTK, Autopsy, bulk-extractor, and Scalpel for analyzing Scientific Linux images, researchers found that both commercial and open-source tools demonstrated different strengths in extracting operating system details, user accounts, web browsing history, and recovering deleted files [19]. This finding highlights that tool selection should be guided by specific investigation requirements rather than general categorization.

Mobile forensics represents another domain in which comparative studies have yielded valuable insights. Recent research comparing Autopsy, Belkasoft X, and Magnet Axiom for analyzing forensic image files from mobile devices operating on Android and iOS systems revealed varying effectiveness across different analytical tasks [24]. These variations underscore the importance of understanding tool capabilities in relation to the specific investigation objectives.

For IoT forensics, open-source tools have shown particular promise due to their adaptability and lower resource requirements. The study evaluating open-source forensic tools for digital evidence collection from various IoT devices has demonstrated their effectiveness and efficiency in conducting standard digital forensic tasks across popular IoT operating systems [25]. This finding is significant given the increasing importance of IoT devices as sources of digital evidence in modern investigations.

Memory forensics represents a specialized area where both open-source and commercial tools offer valuable capabilities. Tools such as Volatility (open source), FTK (commercial), and EnCase (commercial) play important roles in extracting evidence from memory dumps with different strengths in specific analytical tasks [26]. Recent developments have focused on integrating memory forensic capabilities into broader forensic platforms, thereby enhancing investigator efficiency through seamless workflow [21]. Table 1 outlines the comparison between open-source and commercial digital forensic tools, and Table 2 highlights a specific tool comparison.

**Table 1:** Comparative analysis of open-source and commercial digital forensic tools.

Aspect	Open-source tools	Commercial tools
Cost	Free or minimal cost; no licensing fees	High initial investment; recurring licensing fees
Source code access	Fully accessibile, allow customization and validation	Closed limited customization options
Support	Community based, forums and documentation	Profesional, dedicated technical support
Updates	Community driven, potentially irregular	Regular, vendor controlled updates
User interface	Often less refined, steeper learning curve	Sophisticated, user-friendly design
Integration	May require manual integration of multiple tools	Well integrated comprehensive solution
Device support	Variable, may lag for newest devices	Extensive, regularly updated for new devices
Documentation	Community created, potentially less comprehensive	Professional, detailed documentation
Legal Acceptance	Accepted with proper process documentation	Often comes with certifications and legal validation
File Recovery	Effective for standard recovery scenarios	Superior for complex recovery situations
Memory Forensics	Strong capabilities (e.g., Volatility)	Integrated into comprehensive solutions
Mobile Device Analysis	Improving but may lag for newest devices	Comprehensive support for wide device range
Anti-forensic Detection	May struggle with advanced techniques	Often includes specialized anti-forensic detection
IoT Forensics	Flexible; adaptable to various IoT environments	Emerging specialized IoT capabilities
Customizability	Highly customizable; adaptable to specific needs	Limited to vendor-provided options

**Table 2:** Specific tool comparison.

Tool name	Type	Key strengths	Limitations	Best use cases
Autopsy/ TSK	open-source	File system analysis, modular architecture, plugin support	Challenges with encrypted steganography	General forensic analysis, educational environments
Volatility	Open-source	Memory forensics, process analysis, malware detection	Steeper learning curve	Memory dump analysis, malware investigation
Suricata	Open-source	Network traffic analysis, multiprocessing capabilities	Requires networking expertise	Network forensics, intrusion detection
EnCase	Commercial	Comprehensive analysis, strong legal acceptance	High cost, proprietary algorithms	Enterprise investigations, legal proceedings
FTK	Commercial	Advanced data recovery, integrated analysis	Expensive, resource-intensive	Complex investigations, deleted data recovery
Cellebrite UFED	Commercial	Extensive mobile device support, multiple acquisition methods	Very high cost, vendor dependency	Mobile device investigations, law enforcement
Belkasoft X	Commercial	User-friendly interface, strong mobile support	Cost, closed-source limitations	Digital investigations requiring simplified workflow

Several factors influence the selection of digital forensic tools in specific scenarios. Cost is a primary consideration, with open-source tools offering significant advantages for budget-constrained organizations. However, the total cost of ownership should consider factors beyond acquisition costs such as training, support, and maintenance. Commercial tools often provide professional training and support services, potentially reducing the overall implementation time and enhancing investigator efficiency.

Legal admissibility of evidence represents another critical factor. Commercial tools often emphasize compliance with forensic standards and legal requirements, potentially enhancing the credibility of evidence in court proceedings. However, open-source tools can offer advantages in terms of transparency and verifiability, allowing independent validation of forensic processes. Recent research has highlighted the importance of considering legal requirements when selecting forensic tools, emphasizing that both open-source and commercial tools must meet stringent standards for evidence admissibility [3].

The specific requirements of the investigation scenario also significantly influenced tool selection. For specialized investigations involving particular operating systems, comparative studies have shown that different tools exhibit varying levels of effectiveness in extracting relevant evidence [19]. Similarly, for mobile device investigations, tool selection depends on the specific device type, operating system, and data types targeted [23,24].

Organizational capabilities and expertise are additional factors that affect tool choice. Organizations with experienced forensic teams may be better positioned to leverage open-source tools effectively and customize them for specific investigation requirements. Conversely, organizations with limited forensic expertise may benefit from comprehensive solutions and professional support offered by commercial tools.

## Methodology

In this study, the performances of digital forensic tools were validated by testing the repeatability of their analytical results and measuring their error rate in the analysis of digital evidence samples. Thus, the experiments were conducted based on the study by Manson et al. (2007) [26], where a set of test cases was created that simulated various scenarios, such as recovering deleted files, analyzing evidence and extracting data from image files. Similarly, experiments were completed for each test case using both open-source and commercial digital forensic tools, and the results were recorded. Descriptive statistical techniques were used to analyze the data. Descriptive statistics were used to summarize the results of each test case, such as the number of files recovered or the time taken to complete the analysis. This methodology tests the outcome of a hypothesis that ensures the admissibility of digital evidence derived from open-source tools through validation procedures based on the conceptual framework of Ismail et al. (2024) [3]. Details of the instruments, samples, and methods are explained in the following subsections.

The conceptual framework in [3] was developed based on an extensive systematic literature review. This framework is structured around four critical factors that collectively influence evidence admissibility: availability and capability of tools, reliability and integrity of evidence, transparency of tools and established references and standards. It integrates these elements into a cohesive model, where the capability and availability of open-source tools form the foundation, supported by reliability, integrity, and transparency factors that directly impact evidence admissibility in legal contexts. Furthermore, it enables a structured approach to digital forensic investigations using open-source tools, complemented by a preliminary standard operating procedure (SOP) that incorporates three distinct phases: for basic forensic processes, for result validation, and for digital forensic readiness implementation. The framework emphasizes the validation of digital evidence through repeated testing to ensure reliability and accuracy before court presentation, addressing a critical gap in current digital forensic practice.

### Experimental instrument selection

Instrument selection for the study involved the selection of workstations and digital forensic tools.

#### Workstations.

The experiment was conducted at the Digital Forensic Laboratory at the headquarters of the Pharmacy Enforcement Division, Ministry of Health. The experiment was conducted to ensure a controlled environment. There were two (2) Windows base workstations used in the experiment, as stated in Table 3.

**Table 3:** Digital forensic workstation.

Worstation name	Specification	Tool installed
Fred DF Workstation	Intel Core i7-6800k CPU @ 3.40GHz, RAM: 64GB, Windows 10 Pro 64-bit, NVIDIA GeForce GTX 1050 Ti 4GB	FTK
Asus Nitro 5	AMD Ryzen 5 2500U, RAM: 8GB, Windows 10 Pro 64-bit, NVIDIA GeForce GTX 1050 2GB	Autopsy and ProDiscover

#### Commercial digital forensic tools

The list of the tools are as follows:

- AccessData Forensic Toolkit (FTK) Version: 7.5.1.127 is licensed digital forensic software used for analyzing and investigating electronic data. It is a comprehensive tool that can extract data from various sources, such as computers, smartphones, and other electronic devices, and provides a thorough examination of evidence. This tool allows for an in-depth examination of file systems, unallocated space, and volatile memory. FTK also offers various reporting options and can process data in a non-destructive manner, making it ideal for use in forensic investigations.
- Forensic MagiCube (software: ForensicsMaster V6.1.60988) is a licensed portable digital forensic hardware that is used to collect and analyze data from digital devices. It provides a user-friendly interface during the investigation and management of data in a secure and efficient manner onsite at any location. The tool is equipped with various features such as image acquisition, data carving, and file system analysis.

#### Open-source digital forensic tools

The list of the tools are as follows:

- Autopsy Version: 4.19.3 is open-source digital forensic software developed by the Basis Technology Corporation. It is used to analyze and investigate digital evidence to assist in cybercrime investigations, eDiscovery and incident responses. Autopsy provides a graphical user interface and is built on the Sleuth Kit. Autopsy allows digital forensic analysts to examine data from various sources, including hard drives, memory dumps, and removable media in a secure and organized manner. The platform can perform a wide range of forensic tasks, including file system analysis, hash calculation, and keyword search. It also provides the ability to add custom modules, allowing users to extend their functionality to suit specific needs.
- ProDiscover Basic Version: 6.5.0.0 is a tool developed by Technology Pathways LLC. It is used to help investigators and digital forensic analysts analyze and investigate digital evidence in support of criminal investigations, incident response, and eDiscovery. ProDiscover has a graphical user interface and is equipped with features that allow the examination of data from various sources, including hard drives, memory dumps, and removable media. The tool can perform various forensic tasks such as file system analysis, data carving, and hash calculation. It can also search for and identify specific data, such as keywords, and can generate reports to document findings.

### Digital evidence sample preparation

Two (2) sets of digital evidence samples were prepared to test this framework. The sample set was retrieved from the NIST Computer Forensic Reference Dataset (CFReDS). CFReDS is a collection of testable reference sets and a forensic image of simulated digital evidence [27].

Each sample was loaded into a source disk, which was a USB flash drive. The output data are stored on a destination external hard disk. The details of the source and destination disks are listed in Table 4.

**Table 4:** Disk information.

Source disk	Destination disk
Interface: USBeak Model: Kingston DataTraveler 2.0eak Firmware revision: 1.00eak Serial number: 6eak USB Serial number:60A44C426695F0813625E8FEeak Capacity in bytes: 7,747,397,632 (7.7 GB)eak Block Size: 512 byteseak Block Count: 15,131,636	Interface: USBeak Model: Seagate BUP Slimeak Firmware revision: 0004eak Serial number: 00000000NAB60Z1Leak USB Serial number: 00000000NAB60Z1Leak Capacity in bytes: 1,000,204,885,504 (1.0 TB)eak Block Size: 512 byteseak Block Count: 1,953,525,167

The USB flash drive was first forensically cleaned using a Tableu 1.4.1 drive wiper three (3) times to ensure that the source disk was forensically clean to avoid any errors during the testing. Only then will sample images be loaded onto the source disk.

The details of the sample digital evidence are as follows:

- Image 1 is a forensic image of a USB flash drive obtained from NIST CFReDS 2022, set up by the Digital Forensic Research Workshop (DFRWS) as part of a forensic challenge. The image contains multiple types of files, such as text, audio, html, application, Java, and other miscellaneous files. Random types of files are meant to simulate the various contents typically found on digital evidence of a suspect’s disk storage space. The files were extracted from the image and loaded onto the source disk to test the framework during the preservation and collection phases. The hash value of the source disk is calculated using DF tools as a control sample.
- Image 2 is a forensic image of a laptop provided by Digital Corpora. The image is created as part of the M57-Jean scenario, which involves the theft of business documents from a senior executive’s laptop. The M57-Jean scenario was described as: “M57.Biz is a startup business which was hit with a incident involving the leak of personal information of employees online. A private spreadsheet with the social security numbers and salaries was discovered uploaded to one of the company’s main competitors. An image captured from an employee’s laptop disc is preserved. The objective is to how the data was leaked online.”.

### Testing framework

A framework based on the work of Ismail et al. (2024) [3] was tested in this study. The framework was tested using scenarios during the examination and analysis phases. The testing framework was based on the methodologies discussed by Flavien (2014) [28]. The study discussed tool testing through the validation of the results and compared them to a control set. Beckett (2007) [29] defines validation as the process of examining and providing unbiased proof that the forensic outcomes fulfill the requirements for the intended usage. The methodology follows a three (3) step process for validation.

- Define function requirements and assessment metrics.
- Develop and utilize cases and reference sets.
- Run tests and assess metrics.

This method offers a reliable way to assess the reliability of results. This strategy makes the most sense, given that an expected result will be met in certain forensic scenarios while failing miserably for others. The failed results of the evaluation are considered useless and not forensically reliable.

The second test was The National Institute of Standards and Technology (NIST) Computer Forensics Tool Testing (CFTT). The CFTT is a program that provides a systematic and comprehensive approach for evaluating and testing computer forensic tools. The goal of the CFTT is to provide impartial and objective information about the functionality, accuracy, and reliability of computer forensic tools to assist law enforcement, forensic practitioners, and other stakeholders in making informed decisions when selecting tools for their investigations. The testing methodology includes a set of procedures, tools, and criteria that are used to perform a thorough evaluation of the target tool, including its ability to collect, preserve, analyze, and present digital evidence. The results of the testing are reported in a standardized format and are made publicly available, providing valuable information to support tool selection and improve the overall quality and performance of computer forensic tools [29].

**Fig 1:**
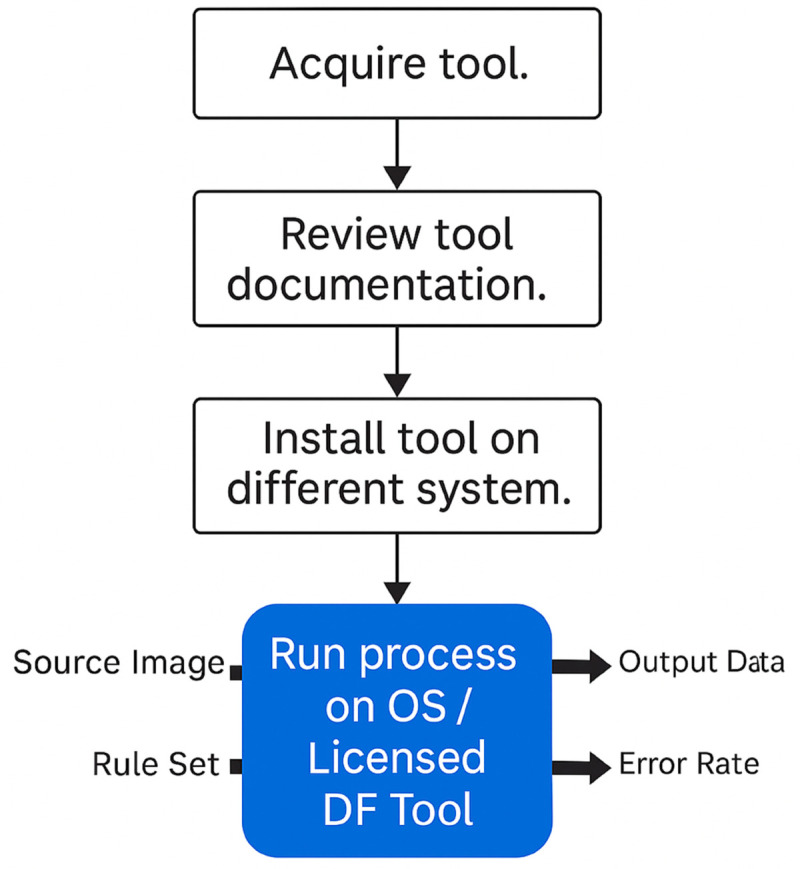
Experiment design framework.

Thus, the experiment was designed and conducted as shown in Fig 1. A forensic image was used as the source image to test different scenarios that analysts usually find during their investigation, which usually involves the preservation, collection, examination, and analysis phases. The rule set is the objective in different phases of the investigation, and tests the capability of the DF tools to achieve the given objectives. The output data are the results obtained from the DF investigation, and the error rate is calculated to validate the result based on hash values and data output.

### Rule set

The rule sets are sets of experimental scenarios based on different stages of digital forensic investigation. The phases are divided into reservation/collection and examination/analysis phases. The framework was tested against a rule set to determine its adaptability to different digital forensic phases in a controlled environment. The experimental scenarios are summarized in Table 5.

**Table 5:** Rule set of the test framework.

Experiment	Sample	Digital forensic phase	Description
Scenario 1	Image 1	Preservation & Collection	Files from image 1 is loaded into the source disk. The objective is to preserve an image of the disk and artifacts is then extracted.
Scenario 2	Image 1	Preservation & Collection	The USB flash drive from experiment 1 is formatted and created an image from the formatted disk. The objective is to recover the lost data due to disk formatting
Scenario 3	Image 2	Examination & Analysis	Examination and analysis of image 2 to achieve the objective of the case based on the M57-Jean scenario.

### Error rate

In digital forensics, error rate refers to the percentage of incorrect results obtained from forensic experiments or analyses. Error rate is an important factor to consider when evaluating the reliability and accuracy of a forensic tool or method. A high error rate can have serious consequences as it may result in false accusations, missed evidence, or incorrect conclusions [28].

There are several sources of errors in digital forensics, including measurement errors, operator errors, and algorithmic errors. Measurement errors occur when the data being collected or analyzed are not accurately represented. Operator error refers to mistakes made by forensic analysts such as incorrect interpretation of data or failure to follow proper procedures. An algorithmic error occurs when the software or tool being used has flaws or bugs that can result in incorrect results.

To minimize the error rate in digital forensics, it is important to follow established protocols and guidelines, use reliable and validated tools and methods, and thoroughly understand the underlying technology and data being analyzed. Additionally, it is important to continuously evaluate and test digital forensic tools and methods to identify and address sources of error.

In this study, the error rate was calculated using Eq (1)

$$Error_{r}ate=\frac{kN\%}{N}$$ (1)

Where N is the artifact acquired from the control, and k is the artifact acquired from the test. Our study chose a 5% threshold to determine the admissibility of evidence produced from digital forensic tools.

### Experimental steps

Three (3) different experiments were performed during the testing phase. The steps for the experiments were as follows:

- Experiment 1
  Step 1: Tools are installed at workstations.
  Step 2: The Source disk containing Image 1 is connected to the workstation through a write blocker.
  Step 3: The destination disk is connected to the workstation.
  Step 4: An image of the source disk is obtained using commercial or open-source tool
  Step 5: Artifacts were collected from the image.
  Step 6: The result is compared with the reference experiment.
- Experiment 2
  Step 1: The source disk from Experiment 1 was formatted using the Windows disk format
  Step 2: The Source disk is reconnected to the workstation using a write blocker.
  Step 3: An image of the source disk is obtained using tools available in workstation.
  Step 4: Recover the deleted data by data carving.
  Step 5: The results are compared to the reference experiment.
- Experiment 3
  Step 1 – The Source disk containing Image 2 is connected to the workstation through a write blocker.
  Step 2: The destination disk is connected to the workstation.
  Step 3: Search for artifacts related to the objective of the case.
  Step 4: Calculate the hash value-acquired artifacts, and compare the result with the reference experiment.

After all the experiments were completed three (3) times each, the results were tabulated and compared to the reference set. If the repeatable results across all the tests achieved, then a pass (P) will be given. Alternatively, if any error or inconsistent in the results, then it is considered as fail (F).

### Results

The control experiment was performed utilizing two (2) commercial tools: the FTK and Forensic Magicube. The results from the control experiment were used as the reference sample in the experiment.

#### Control experiment

For FTK tool:

- Control Experiment 1.
  Forensic images were captured from source disks. This process was repeated for a total of three tests. All three images were verified using the same MD5 and SHA1 hash values, as listed in Table 6. A total of 175 artifacts (126489439 bytes) were extracted from each image. All the files were verified, and no errors were found.
- Control Experiment 2.
  The results from the preservation using FTK Imager and data carving for three (3) tests are summarized in Table 7. Forensic images were captured using a formatted source disk. The process was repeated, with a total of three tests being carried out. All three images were verified using the same MD5 and SHA1 hash values. A total of 112 artifacts (138018816 bytes) were successfully obtained from each image. All the files were verified, and no errors were found.
- Control Experiment 3.
  Examination and analysis were performed on the images. An .X03LS Excel spreadsheet was found containing the leaked information. The file was created on 20.07.2008, which consists of the reported incident date. The files were then extracted and presented as evidence. This process was repeated and the results were verified. No errors were found. The extracted artifact was a file named m57biz.xls, and the hash value was verified, as listed in Table 8.

**Table 6:** Result of Control Experiment 1 using FTK.

No of test	Image hash value	Bytes	No of Artifact extracted	Duration (Min)
1	MD5: 41876dfd6e6e6ee3501118f7bb887a2aeak SHA1: b1a44ef8e5f23e933f0561cc8dc1700edd0ba3bf	126489439	175	4.15
2	MD5: 41876dfd6e6e6ee3501118f7bb887a2aeak SHA1: b1a44ef8e5f23e933f0561cc8dc1700edd0ba3bf	126489439	175	4.1
3	MD5: 41876dfd6e6e6ee3501118f7bb887a2aeak SHA1: b1a44ef8e5f23e933f0561cc8dc1700edd0ba3bf	126489439	175	4.1

**Table 7:** Result of Control Experiment 2 using FTK.

No of test	Image hash value	Bytes	No of Artifact extracted	Duration (Min)
1	MD5: 41876dfd6e6e6ee3501118f7bb887a2aeak SHA1: b1a44ef8e5f23e933f0561cc8dc1700edd0ba3bf	138018816	112	4.6
2	MD5: 41876dfd6e6e6ee3501118f7bb887a2aeak SHA1: b1a44ef8e5f23e933f0561cc8dc1700edd0ba3bf	138018816	112	4.6
3	MD5: 41876dfd6e6e6ee3501118f7bb887a2aeak SHA1: b1a44ef8e5f23e933f0561cc8dc1700edd0ba3bf	138018816	112	4.6

**Table 8:** Result of Control Experiment 3 using FTK.

No of test	Image hash value	Bytes	No of Artifact extracted
1	MD5: e23a4eb7f2562f53e88c9dca8b26a153eak SHA1: 55638af43dddd0f1ff8cd4dab73b2979ac5be8b1	291840	1
2	MD5: e23a4eb7f2562f53e88c9dca8b26a153eak SHA1: 55638af43dddd0f1ff8cd4dab73b2979ac5be8b1	291840	1
3	MD5: e23a4eb7f2562f53e88c9dca8b26a153eak SHA1: 55638af43dddd0f1ff8cd4dab73b2979ac5be8b1	291840	1

For Forensic MagiCube

- Control Experiment 1.
  The results from the preservation using Forensic MagiCube of the three (3) tests are summarized in Table 9. A forensic image was captured from the source disk by using a Forensic MagiCube. The process was repeated, with a total of three tests carried out. All three images were verified using the same MD5 and SHA1 hash values. A total of 175 artifacts (126489439 bytes) are extracted from each image. All the files were verified, and no errors were found. This result was consistent with that of FTK.
- Control Experiment 2.
  The results from the preservation using the FTK Imager and data carving of the three (3) tests are summarized in Table 10. A forensic image was captured from a formatted source disk by using this tool. The process was repeated, with a total of three tests carried out. All three images were verified using the same MD5 and SHA1 hash values. A total of 112 artifacts (138018816 bytes) were successfully extracted from each image. All the files were verified, and no errors were found. These results are also consistent with the FTK results.
- Control Experiment 3.
  The examination and analysis processes were also conducted, similar to FTK. As with the FTK results, all three (3) results were consistent without any errors.

**Table 9:** Result of Control Experiment 1 using Forensic MagiCube.

No of test	Image hash value	Bytes	No of Artifact extracted	Duration (Min)
1	MD5: 41876dfd6e6e6ee3501118f7bb887a2aeak SHA1: b1a44ef8e5f23e933f0561cc8dc1700edd0ba3bf	126489439	175	6.08
2	MD5: 41876dfd6e6e6ee3501118f7bb887a2a SHA1: b1a44ef8e5f23e933f0561cc8dc1700edd0ba3bf	126489439	175	6.07
3	MD5: 41876dfd6e6e6ee3501118f7bb887a2aeak SHA1: b1a44ef8e5f23e933f0561cc8dc1700edd0ba3bf	126489439	175	6.07

**Table 10:** Result of Control Experiment 2 using Forensic MagiCube.

No of test	Image hash value	Bytes	No of Artifact extracted	eak Duration (Min)
1	MD5: 41876dfd6e6e6ee3501118f7bb887a2aeak SHA1: b1a44ef8e5f23e933f0561cc8dc1700edd0ba3bf	138018816	112	6.9
2	MD5: 41876dfd6e6e6ee3501118f7bb887a2a SHA1: b1a44ef8e5f23e933f0561cc8dc1700edd0ba3bf	138018816	112	6.7
3	MD5: 41876dfd6e6e6ee3501118f7bb887a2a SHA1: b1a44ef8e5f23e933f0561cc8dc1700edd0ba3bf	138018816	112	6.7

#### Experiment with open-source digital forensic tool

**Fig 2:**
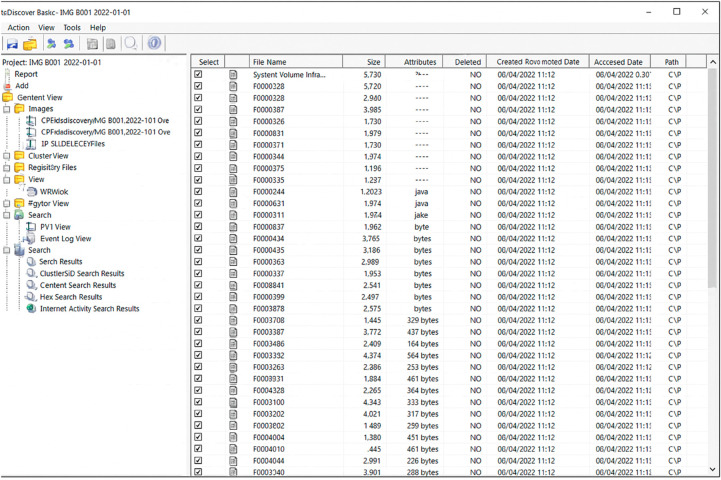
Image acquired using ProDiscover Basic in Experiment 1.

This section presents and discusses the results of an experiment conducted using open-source digital forensic tools. The results of the experiment were compared with those of the control experiment. The findings are then analyzed and discussed to determine the viability of the developed open-source framework. The following is the result for both Autopsy and ProDiscover Basic tools:

- Experiment 1.
  Three forensic images were successfully acquired using both Autopsy and ProDiscover. All three images were verified using the same MD5 and SHA1 hash values, as listed in Table 11. A total of 175 artifacts (126489439 bytes) were extracted from each image. All the files were verified, and no errors were found. Both tools completed the task accurately. However, in terms of duration, ProDiscover can provide a shorter time for the task. Fig 2 presents the image acquired using ProDiscover Basic in Experiment 1.
- Experiment 2.
  Autopsy has successfully acquired three images from the formatted source disk. In total, 112 deleted files (138018816 bytes) were recovered from each image. No error was found in the dataset. Similarly, ProDiscover acquires three images from the formatted source disk. However, this tool lacks the capability to recover the lost data in the image. Data recovery was completed using Autopsy instead by importing the image created by this tool. No errors were found after data verification.
- Experiment 3.
  The artifact, a file named m57biz.xls was found in image 2 using Autopsy. The examination and analysis processes were repeated, and hash values were verified. No errors are observed in the data. On the another hand, Experiment 3 for ProDiscover could not be completed. It is due to the tool documentation that it can only read ProDiscover (.EVE) and UNIX (.DD) image.

**Table 11:** Result of Experiment 1 using Autopsy and ProDiscover.

No of test	Image hash value	Bytes	No of Artifact extracted	Duration- Autopsy (Min)	Duration ProDiscover (Min)
1	MD5: 41876dfd6e6e6ee3501118f7bb887a2aeak SHA1: b1a44ef8e5f23e933f0561cc8dc1700edd0ba3bf	126489439	175	22.5	18.5
2	MD5: 41876dfd6e6e6ee3501118f7bb887a2aeak SHA1: b1a44ef8e5f23e933f0561cc8dc1700edd0ba3bf	126489439	175	22.5	11.9
3	MD5: 41876dfd6e6e6ee3501118f7bb887a2aeak SHA1: b1a44ef8e5f23e933f0561cc8dc1700edd0ba3bf	126489439	175	18.7	14.2

## Discussion

The study by Ismail et al. (2024) [3] identified four (4) issues relating to digital evidence produced by DF tools that were most discussed in existing studies. The factors affecting the admissibility of digital evidence produced by open-source digital forensic tools can be summarized as follows:

- Capability and availability of open-source digital forensic tools for investigation.
- Reliability and integrity of digital evidence produced by open-source digital forensic tools.
- Transparency of open-source digital forensic tools used during investigation.
- The lack of references and standards relating to the use of open-source digital forensic tools in an investigation.

### Capability and availability of open-source digital forensic tools

In this study, we addressed the capabilities and reliability of open-source digital forensic tools compared with proprietary tools for producing forensically sound evidence. The results from the experiment above are summarized in the Tables 12, 13 and 14 to show the capabilities and consistency of the results from open-source digital forensic tools compared to the results of the control experiment.

**Table 12:** Result comparison of Experiment 1.

Tool	Result validation			Average	Error
	(P-Pass, F-Fail)			Duration	Rate
	Test 1	Test 2	Test 3	(min)	%
FTK	P	P	P	4.1	0
Magicube	P	P	P	6.1	0
Autopsy	P	P	P	21.2	0
ProDiscover	P	P	P	7.4	0

**Table 13:** Result comparison of Experiment 2.

Tool	Result validation			Average	Error
	(P-Pass, F-Fail)			Duration	Rate
	Test 1	Test 2	Test 3	(min)	%
FTK	P	P	P	4.6	0
Magicube	P	P	P	6.8	0
Autopsy	P	P	P	21.5	0
ProDiscover	P	P	P	14.9	0

**Table 14:** Result comparison of Experiment 3.

Tool	Result validation (P-Pass, F-Fail)			Error Rate %
	Test 1	Test 2	Test 3	
FTK	P	P	P	0
Magicube	P	P	P	0
Autopsy	P	P	P	0

The experiment showed that all the tested tools, Autopsy and ProDiscover, were able to perform all the tasks involved in the DF investigation, including preservation, collection, examination, and analysis. The exception was the ProDiscover tool, which was unable to recover the lost data in a formatted disk directly from the tool itself. However, the tool successfully acquired the image and then utilized Autopsy data, which was recovered. All experiments produced consistent, repeatable results, which is one of the requirements for validating digital evidence based on the NIST standard. The result is validated by comparing the hash value, whereby tests with the same hash value are determined as P-Pass, and any changes in the hash value are determined as F-Fail.

The developed open-source digital forensic framework fulfills parts of The Daubert Standard for evaluating the reliability and validity of digital forensic evidence. As shown in the experiments, all results produced by the open-source digital forensic tool were testable and showed repeatable results.

In addition to reliability and capability, the availability of these tools ready for use in digital forensic investigations is also important. There are a wealth of open-source digital forensic tools capable of undergoing all types of processes in the types of digital evidence being handled in a case. In this study, Autopsy and ProDiscover were selected as the tested tools because both of these tools have multifunctional operations, such as creating a disk image, keyword searches, email analyses, registry analysis, and many more, which are comparable to the commercial digital forensic tool. These functionalities are critical in all aspects of an in-depth forensic investigation to collect, preserve, and analyze digital evidence. A comparison of these tools is presented in the Table 15.

**Table 15:** Tool comparison.

Category	FTK	Forensiceak MagiCube	Autopsy	ProDiscover
Forensic Type	Computer	Computer	Computer,eak Mobile Phone	Computer
Cost	Paid Licensed	Paid Licensed	Free Open-Source	Free Open-Source
Tool type	Software	Hardware	Software	Software
File Format	RAW, SMART,eak E01, AFF	RAW, 001,eak E01	RAW, IMG, DD,eak BIN, 001, E01,eak VHD, etc.	EVE, DD
Multiuser Function	Limited	No	Yes	Yes
Hash Function	MD5, SHA1	MD5, SHA1	MD5, SHA1,eak SHA256	MD5
Image acquisition	Yes	Yes	Yes	Yes
Shows hasheak for individualeak file	Yes	Yes	Yes	Yes
Can verifyeak image integrity	Yes	Yes	Yes	Yes
Find deleted files	Yes	Yes	Yes	No
Recover deleted files	Yes	Yes	Yes	No
Find encrypted files	Yes	Yes	Yes	Yes
Includes HEX leveleak viewer	Yes	Yes	Yes	Yes
Organizes files intoeak predetermined categories	Yes	Yes	Yes	Yes
Shows image gallery	Yes	Yes	Yes	No
Shows fileeak modified/accessed/eak created times	Yes	Yes	Yes	Yes
Provides a log file ofeak investigation activity	Yes	Yes	Yes	Yes
Process speed	Fastest	Fast	Slowest	Slow

### Reliability and integrity of digital evidence produced by open-source digital forensic tools

The reliability and integrity of digital evidence refer to the trustworthiness and accuracy of digital data that are collected and used as evidence in legal or investigative contexts.

Reliability refers to the consistency and dependability of digital evidence. Reliable digital evidence is evidence that has been properly collected and preserved and that is not corrupted or altered in any way. This means that digital evidence should remain unmodified and unchanged from the time it was collected to the time it was used as evidence [31]. Integrity refers to the completeness and accuracy of the digital evidence. Digital evidence must be authentic and represent true and accurate representations of the original data. This means that digital evidence should not be tampered with or altered in any way and that it should provide a complete and accurate representation of the original data [32].

Both reliability and integrity are critical considerations in digital forensics because the accuracy and credibility of digital evidence can have a significant impact on the outcome of legal or investigative proceedings. To ensure the admissibility of digital evidence, the open-source digital forensic tool used during the collection, preservation, and analysis of digital evidence must be accurate without compromising the integrity of the digital evidence.

In the experiment conducted above, we demonstrated that the open-source digital forensic tools used in the experiments were able to produce accurate and repeatable results. The integrity of the image and files was also maintained after the open-source digital forensic tool completed the processes during the experiment. This was demonstrated by the checksum hash value of the image and files acquired during the test, with a 0% error rate.

### Transparency of the open-source digital forensic tools usage during investigation

Transparency refers to the openness and accessibility of information regarding the design, implementation, and functioning of a digital forensic tool. In the context of open-source digital forensic tools, transparency refers to the availability of source code, documentation, and other related information [33].

Open-source digital forensic tools are tools whose source code is publicly available, allowing anyone to view, use, modify, or distribute code. This level of transparency enables digital forensic practitioners, researchers, and other stakeholders to understand exactly how the tool works, which can increase trust and confidence in the tool’s results [29].

Transparency is an important consideration in digital forensics as it helps ensure that the methods and techniques used in a digital forensic investigation are transparent and open to scrutiny. This can help avoid potential biases or inaccuracies in the results and increase the reliability and credibility of the digital evidence obtained. This is an advantage of open-source digital forensic tools such as Autopsy and ProDiscover, in which source codes are readily available to be scrutinized without bias. In contrast to their proprietary counterparts, the source code of tools is more often than not a closely guarded business secret [34].

In the process of selecting the open-source digital forensic tools, the documentation regarding Autopsy and Prodiscover was readily available to be read and reviewed. The documentation clearly explains and details the functionality of these tools.

The transparency of the documentation of these open-source digital forensic tools also demonstrates their update frequency and available technical support. As with other OS software, the updates and technical support for open-source digital forensic tools are mostly community driven. Most documentation and support can be obtained from GitHub and other related public forums. Tina et al. (2020) [39] in their study stated that open-source digital forensic tools often suffer from a lack of proper support, documentation, and safety updates to software. Therefore, it is integral to properly review open-source digital forensic tool documentation to determine the selection and tool validation policy of these tools.

### Lack of references and standards related to the use of open-source digital forensic tools in investigation

The lack of well-established and widely recognized guidelines and best practices for using open-source digital forensic tools can make it challenging for DF analysts to select the most suitable tool for a specific investigation. It also presents a challenge to ensure the reliability and validity of the results intended for presentation in court [35]. These challenges can significantly impact the credibility and reliability of results obtained using open-source digital forensic tools. For example, the absence of established protocols and methodologies can make it difficult for digital forensic analysts to ensure that the obtained results are accurate and that the evidence obtained is admissible in a court of law. Thus, certain legal and technical standards must be met for digital evidence to be admissible in the court of law. Antwi-Boasiako and Hein Venter (2017) [36], discussed the five (5) general rules of evidence that determine the admissibility of digital evidence:

- Relevance: Evidence must be relevant to the facts of the case in order to be admissible. This means that evidence must have a direct bearing on the issue being litigated and must help to prove or disprove a fact in dispute.
- Authenticity: Evidence must be authentic in order to be admissible. This means that the evidence must be shown to be what it purports to be and must not have been altered or tampered with.
- Completeness: Evidence must be both complete and admissible. This means that the evidence must provide a full and accurate picture of the facts of the case and must not be misleading.
- Reliability: Evidence must be reliable and admissible. This means that the evidence must have been collected, preserved, and processed in a manner that ensures its accuracy and integrity and that the methods used to collect and analyze the evidence are reliable and trustworthy.
- Credibility: Evidence must be credible and admissible. This means that evidence must support a reasonable belief or conclusion and must not be based on speculation, conjecture, or unreliable sources.

The rules of evidence are a set of legal principles that dictate what evidence is admissible in the court of law. These rules are designed to ensure that the evidence presented in court is reliable, relevant, and credible and that the legal process is fair to all parties involved. These rules of evidence are important because they help ensure that the legal process is fair and decisions are based on the best and most trustworthy evidence available. They also help protect the rights of all parties involved and ensure that the legal system operates efficiently and effectively.

The experiments demonstrated that the digital evidence produced by the open-source digital forensic tools complied with the requirements described in the rules of evidence. The results produced from the open-source digital forensic tools were accurate and validated and were comparable to the control reference results using commercial tools. Thus, the open-source digital forensic tools Autopsy and ProDiscover were able to produce digital evidence that was relevant, authentic, complete, reliable, and credible.

In the Malaysian judiciary system, there is still an acceptable reference or standard for the use of the open-source digital forensic tool to produce a piece of admissible evidence. Currently, the admissibility of digital evidence is governed by the Evidence Act of 1950 and the Rules of Court of 2012. These laws and regulations provide guidelines for digital evidence collection, preservation, and admissibility in legal practice.

Under the Evidence Act 1950, digital evidence is admissible as long as it is relevant to the matter in question and is not excluded by any provision of the Act. Digital evidence can be obtained in the form of electronic records, printouts, or other electronic storage devices. Section 90A Evidence Act and Order 24 Rules of Court 2012 also provide guidelines for the admissibility of digital evidence. These rules require that digital evidence be accompanied by a certificate of authenticity, which is a written statement certifying the authenticity of digital evidence. The certificate of authenticity must be signed by the person who collects and preserves the digital evidence, and must specify the method used to collect and preserve the evidence. Therefore, analysts must prepare and equip themselves with the proper knowledge and expertise of the open-source digital forensic tool used in the investigation. Analyst competency and training are addressed in the digital forensic readiness plan. Analysts must be able to present themselves as expert witnesses to their digital forensic reports in court.

## The open-source digital forensic tools framework

When dealing with digital evidence, numerous complications can disrupt and harm the credibility of the digital evidence produced even by a licensed digital forensic tool. Furthermore, the admissibility of digital evidence produced by open-source digital forensic tools is poorly documented by any court of law in Malaysia. The lack of well-documented cases utilizing open-source tools in legal proceedings may discourage the reliability of the digital evidence produced by open-source digital forensic tools.

However, in the United States, there are several cases in which digital evidence produced by open-source digital forensic tools has been successfully used in legal proceedings. According to Rochester, N.Y., a man was convicted of having child pornography. Investigators found incriminating evidence on the offender’s digital device using FieldSearch. Using the report and evidence produced by FieldSearch, the offender was charged in court and sentenced to prison [37]. Therefore, it is possible to implement and practice the use of open-source digital forensic tools to produce reliable and acceptable evidence in the Malaysian judiciary system.

To ensure the admissibility of digital evidence, critical elements such as the accuracy, authenticity, and objectivity of said digital evidence need to be preserved, regardless of which tools are being utilized in an analysis. Consequently, validating the results produced by an open-source digital forensic tool is essential in the digital forensic process. According to the National Institute of Standards and Technology (NIST), digital evidence must be repeatable and reproducible to be accepted in legal proceedings [40].

**Fig 3:**
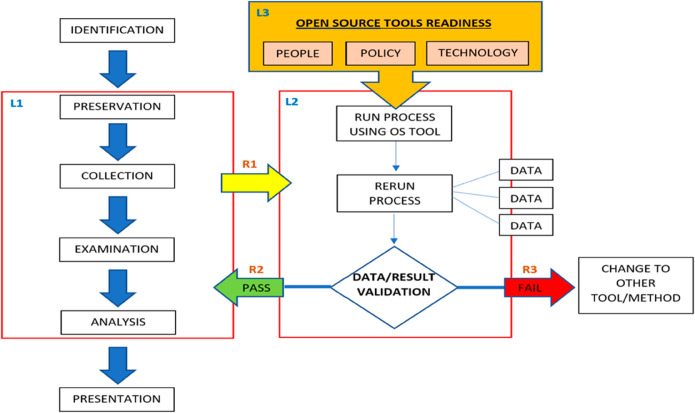
Open-source digital forensic tool framework.

Therefore, based on the findings of the experiments and discussion, we developed a framework for using open-source digital forensic tools for investigation. The framework for open-source digital forensic tools can be described as a step-by-step approach that establishes procedures for forensic analysis of digital evidence using these tools for it to be admissible in court. The open-source digital forensic tool framework is presented in Fig 3.

The proposed framework combines the three (3) phases in the DF investigation process. The L1 phase is where basic DF processes, such as preservation, collection, examination, and analysis, are performed (DFRW 2006). L2 validates the results obtained from open-source digital forensic tools. Finally, L3 is the DFR plan for implementing the OS DF tools. R1 is the point at which an analyst decides to use any open-source digital forensic tool during the preservation, collection, examination, or analysis processes in L1. During L2, the analyst will use open-source tools to fulfill their forensic objectives. The results obtained from these tools were then repeated at least three (3) times and validated by comparing their accuracy and repeatability. R2 is the point at which the validation process passes, and the analyst continues with the process in L1. Otherwise, if the validation process fails, proceed to point R3 and the result is considered inadmissible. In such cases, analysts should consider other tools or methods. The L3 phase describes the readiness requirements for an organization to implement open-source digital forensic tools in investigations.

## Open-source digital forensic readiness (DFR)

Digital forensic readiness (DFR) recognizes an organization’s level of competence in gathering, maintaining, safeguarding, and analyzing digital evidence for use in legal proceedings, disciplinary proceedings, employment tribunals, and courts of law. Brian et al. (2003) [38] described DFR as a continuous activity to ensure that DF operations and infrastructure inside the organization can support an investigation effectively before and after any case.

The open-source digital forensic tool DFR is the set of preparations required by an organization to effectively use open-source digital forensic tools in investigations. Therefore, the objective of DFR for open-source tools is as follows:

- Obtain legally admissible evidence without interfering with organizational processes.
- To allow investigations to be carried out at a cost appropriate to the severity of the incident.
- To ensure that evidence has a favorable influence on the results of court proceedings.
- To avoid any disruption of services by keeping investigations in a minimal but effective manner.

As shown in L3, the four (4) core components are critical to DFR, which includes people, organization policy, and technology. The people component encompasses the training and hiring of skilled analysts, segregation of roles, and security training and awareness campaigns. DFR requires the establishment of a competent expert analyst to securely acquire legally admissible evidence using an open-source digital forensic tool. The second component of policy details the organization’s policies, including policies on digital forensic processes, training, and legal requirements to assist the use of open-source digital forensic tools in the organization’s investigation. The final component of technology includes determining the best open-source tool to use in order to avoid and detect any related issues to facilitate the organization’s digital forensic activities. Therefore, it is essential for digital forensic organizations to develop DFR plans with the introduction of open-source tools today and in the future.

## Advantages and disadvantages of the framework

The advantages of the proposed framework increase the admissibility of the evidence resulting from open-source digital forensic tools by repeating and validating the results. As Malaysia’s judiciary system is yet to set guidelines or standards on the use of open-source tools, arguments could be made by the analyst that the produced evidence is admissible based on the standard set by NIST. In addition, the DFR model incorporated into the framework allows law agencies to plan better and employ open-source tools in their current and future investigations.

However, the framework only validates the results based on repeatability rather than reproducibility. Reproducibility is contingent on dependable tools and methods trusted by the courts. Utilizing licensed tools to validate the reproducibility of the results will be counterproductive to the objective of this study. In addition, analysts will be less likely to adopt this framework if there is any further increase in their workload owing to the increased time and steps in the digital forensic process

## Conclusions

This study validated and enhanced the conceptual framework to ensure the legal admissibility of digital evidence acquired through open-source forensic tools. Through rigorous experimentation comparing open-source tools with commercial alternatives, we demonstrated that open-source solutions can consistently produce reliable and repeatable results with verifiable integrity. The developed three-phase framework effectively addressed critical admissibility factors, including tool capability, evidence reliability, process transparency, and adherence to legal standards. Our empirical findings satisfied the Daubert Standard requirements by establishing repeatability, methodological transparency, and minimal error rates. The integration of Digital Forensic Readiness planning further enhances organizational capability to deploy open-source solutions without compromising evidence integrity. Although our framework demonstrates significant advantages in democratizing access to forensically sound investigative capabilities, it primarily validates results through repeatability rather than full reproducibility, which represents an area for future research. Additional work should focus on developing jurisdiction-specific implementation guidelines and expanding validation across diverse forensic scenarios, particularly emerging technologies, such as IoT and cloud environments. This study ultimately challenges the preference for costly commercial solutions in legal proceedings, while providing resource-constrained organizations with methodologically sound approaches that maintain the evidentiary standards necessary for judicial acceptance.
